# Organ-to-Organ Communication: A *Drosophila* Gastrointestinal Tract Perspective

**DOI:** 10.3389/fcell.2017.00029

**Published:** 2017-04-03

**Authors:** Qiang Liu, Li Hua Jin

**Affiliations:** Department of Genetics, College of Life Sciences, Northeast Forestry UniversityHarbin, China

**Keywords:** *Drosophila*, gastrointestinal tract, interorgan communication, signaling network, organismal homeostasis, innate immune system

## Abstract

The long-term maintenance of an organism's homeostasis and health relies on the accurate regulation of organ-organ communication. Recently, there has been growing interest in using the *Drosophila* gastrointestinal tract to elucidate the regulatory programs that underlie the complex interactions between organs. Data obtained in this field have dramatically improved our understanding of how organ-organ communication contributes to the regulation of various aspects of the intestine, including its metabolic and physiological status. However, although research uncovering regulatory programs associated with interorgan communication has provided key insights, the underlying mechanisms have not been extensively explored. In this review, we highlight recent findings describing gut-neighbor and neighbor-neighbor communication models in adults and larvae, respectively, with a special focus on how a range of critical strategies concerning continuous interorgan communication and adjustment can be used to manipulate different aspects of biological processes. Given the high degree of similarity between the *Drosophila* and mammalian intestinal epithelia, it can be anticipated that further analyses of the *Drosophila* gastrointestinal tract will facilitate the discovery of similar mechanisms underlying organ-organ communication in other mammalian organs, such as the human intestine.

## Introduction

The physiological systems of various multicellular organisms that communicate with each other are of significance for the regulation of homeostasis under varying environmental conditions (Nässel et al., 2013; Droujinine and Perrimon, 2016). The close spatial association between organs within an open circulation correlates with multiple aspects of development, fecundity, growth, metabolic homeostasis, stress responses, and lifespan, the disruption of which underlies the loss of the optimal or steady state (Amcheslavsky and Ip, 2012; Droujinine and Perrimon, 2013). How an organism instructs this process of communication is a question of great complexity.

An excellent model to explore organ-to-organ communication is the *Drosophila* gastrointestinal tract (GI tract). The gastrointestinal mono-layered tube, as a physical barrier, possesses a range of critical defense strategies to ensure organismal health (Royet, 2011). The source of the signals with respect to intestinal homeostasis is not confined to the GI tract. For example, the integral parts of the GI tract, such as the intestinal trachea and enteric neurons, also act as part of the niche to affect the steady state (Kux and Pitsouli, 2014). Likewise, neighbors adjust their performances based on gut-expressed signals.

During the past few years, our comprehension of the mechanisms underlying organ-organ communication has expanded considerably. This review briefly summarizes the integrated molecular network regarding the links between the *Drosophila* GI tract and crucial neighbors, followed by a discussion of the potential value of these intriguing associations in fundamental biological processes, including the maintenance of homeostatic equilibrium.

## An overview of the GI tract

The anatomical details of the adult *Drosophila* GI tract are relatively well known. The adult GI tract is traditionally divided into three discrete domains, the foregut, the midgut and the hindgut, containing the five best-understood stem cells related to turnover (Charroux and Royet, 2010; Zeng et al., 2013). Two studies have investigated sub-specialization to date (Buchon et al., 2013; Marianes and Spradling, 2013). The absorptive enterocytes (ECs) and secretory enteroendocrine cells (EEs) are the main cell types along the GI tract (Lucchetta and Ohlstein, 2012). A short and narrow segment, known as the copper cell region (CCR), is located in the middle (Li H. et al., 2016).

**Figure 1:**
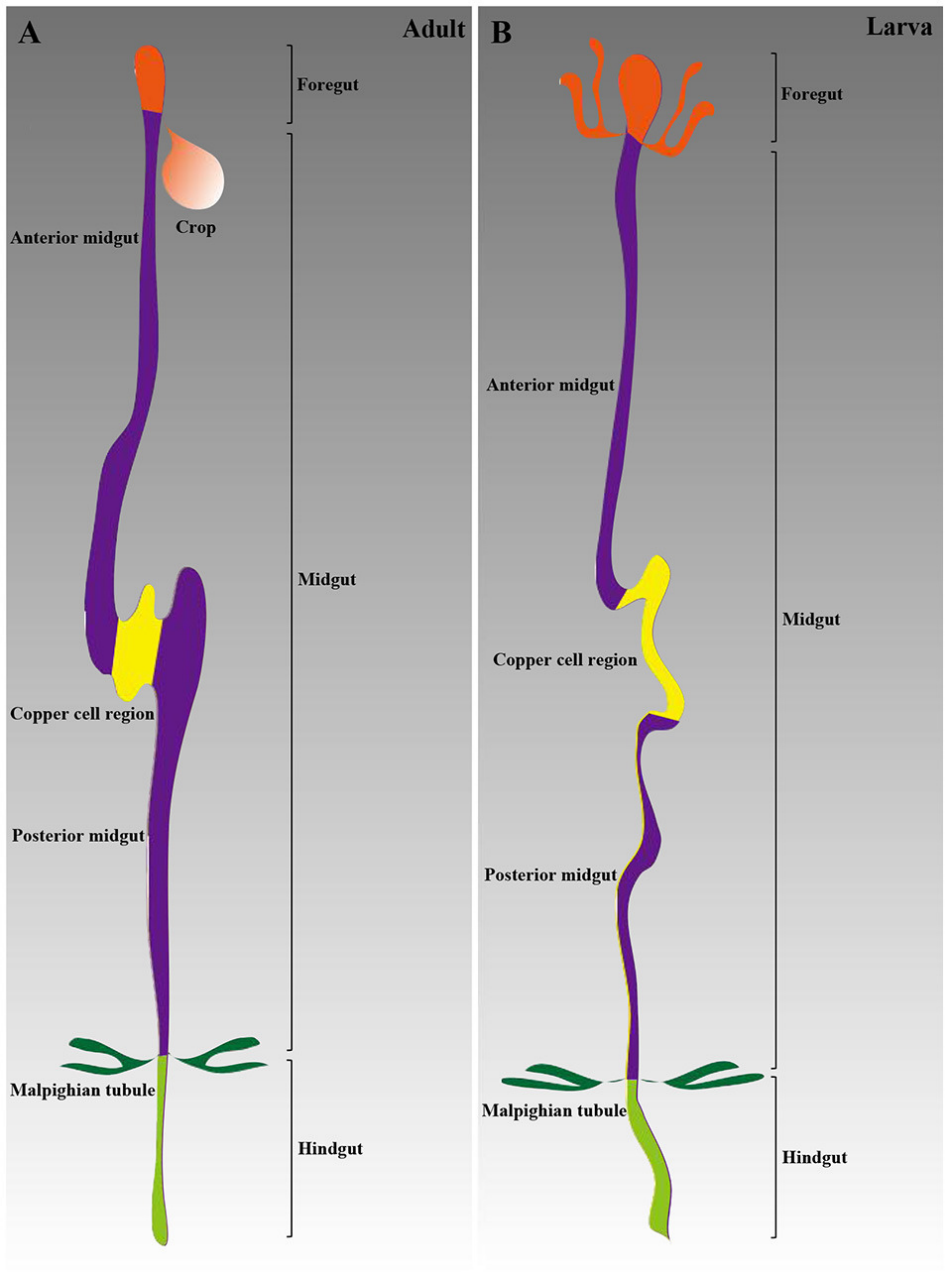
**Schematic of the ***Drosophila*** GI tract anatomy. (A)** The adult GI tract is composed of three main parts: the foregut, the midgut, and the hindgut. The midgut includes three sections: the anterior midgut, the CCR, and the posterior midgut. **(B)** The larval GI tract structure is similar to that in adults.

Similar to adults, the larval midgut has three segments based on the acid-secreting middle segment (Shanbhag and Tripathi, 2009). The gastric cecum is a special segment that does not exist in the adult GI tract. Additionally, the level of cellular transport processes in the larval epithelium is higher than that in adults (Shanbhag and Tripathi, 2009). Functional diversification in subsections of the larval midgut has been analyzed by Harrop et al. (2014). A summary of the GI tract structures of both adults and larvae is provided in Figure 1.

## Good neighbors

The primary concern of studies investigating gut-neighbor axes is to validate the existence of peripheral organs and tissues that communicate with the GI tract. There is now substantial evidence suggesting that several non-gastrointestinal organs and tissues interact with the GI tract to ensure the optimal state of the organism, including the following: (1) One good neighbor is the hemocytes, which are associated with the phagocytosis and encapsulation of invading pathogens. Only three types of differentiated cells, namely plasmatocytes, crystal cells and lamellocytes, can be distinguished (Meister, 2004; Crozatier and Meister, 2007). (2) Sensing and responding to nutrients with the synthesis and release of energy are considered the function of the “liver,” termed the fat body, which interacts with the GI tract to meet different physiological needs (Sousa-Nunes et al., 2011). (3) The enteric neurons that innervate the GI tract were summarized in a recent report showing the gastrointestinal roles of the nervous system in the control of intestinal physiology (Kuraishi et al., 2015). (4) Of particular interest are the insulin-producing cells (IPCs) of the brain, which are thought to regulate growth, metabolism, and stress responses through the production of different insulin-like peptides (Dilps) (Nässel et al., 2013). (5) The GI tract is surrounded by smaller tubes named tracheoles from the tracheal system, which is responsible for delivering oxygen to the epithelium and expelling carbon dioxide, achieving tissue gas exchange and meeting the oxygen needs of intestinal cells (Kux and Pitsouli, 2014). (6) The GI tract is surrounded by circular and longitudinal visceral muscles (VMs), which facilitate the mixing, grinding, and pushing forward of ingested food (Royet, 2011). (7) Within the hemocoelic body cavity, there is an excretory and osmoregulatory organ known as the Malpighian tubules (MTs), which perform a series of functions, such as excreting metabolic waste (Affolter and Barde, 2007). (8) Ovarian function and maintenance require multiple signals from peripheral organs. Each female has a pair of ovaries, and each ovary contains 15–20 ovarioles that consist of a series of egg chambers (Kirilly and Xie, 2007). Other organs that signal to others include the lymph gland, salivary gland, retina, skeletal muscles, and prothoracic gland. In the following, we will discuss recent evidence supporting the involvement of organ-to-organ communication strategies in the regulation of organismal health.

## Organ-to-organ communication

The first integrated ideas discussed in this review focus on the fundamental principles concerning the links between organs in adults, which correlate with the homeostatic regulation of metabolism, physiology and aging and are currently under intense investigation. We will also discuss the larval aspects of interorgan communication. Although an exhaustive summary of all the data in these fields would be impossible, this review will highlight emerging trends.

## A tale in the adult body cavity

### Gut-neighbor axes

#### Hemocytes

First, we present the “state of the art” on the study of hemocyte-gut communication models. Exploring the gut-hemocyte axis has been a recent focus of interest. Breakthrough discoveries include the identification of hemocyte-expressed messages as dedicated regenerative inflammatory factors that facilitate regeneration after intestinal damage. To understand the functional roles of hemocyte-gut communication, we need to consider the location of hemocytes within the adult body cavity. Indeed, most hemocytes are concentrated within the middle of the midgut, several individual hemocytes also appear close to the intestinal epithelium. Jasper H and co-workers have shed light on the temporal sequence of interactions between hemocytes and intestinal cells during pathogen infection (Ayyaz et al., 2015). Hemocyte-expressed Decapentaplegic (Dpp) triggers Saxophone (SAX) and Smad on X (SMOX) activation in the epithelium during the early phase of infection, contributing to intestinal stem cell (ISC) proliferation; re-establishing ISC quiescence requires the activation of Thickvein (Tkv) and MAD. Adults with hemocyte loss are sensitive to infection (Ayyaz et al., 2015). Further studies have rapidly progressed, spurred by Lemaitre B's laboratory (Chakrabarti et al., 2016). Specifically, hemocyte-expressed Unpaireds (Upds) are relevant to ISC mitogenesis after infection, emphasizing a role for hemocytes in intestinal regeneration. It is notable that hemocyte-mediated intestinal regeneration was thoroughly illuminated by the above two reports, based on a model of the direct introduction of bacteria into the enteric cavity. Therefore, it will be interesting to discover the cues by which the pathogen-infected intestine signals to hemocytes, contributing to its regeneration. Additionally, a recent study mapped the apoptotic caspase pathway in the intestine after systemic wounding (Takeishi et al., 2013). Wounding-induced reactive oxide species (ROS) trigger apoptotic caspase pathway activation in ECs, which is responsible for suppressing the production of systemic lethal factors (Takeishi et al., 2013). Caspase activity loss in ECs causes increased levels of systemic lethal factors in hemolymph, enhancing the sensibility of flies to non-infectious stresses (Takeishi et al., 2013). Together, these studies suggest that hemocyte-gut communication acts as a critical strategy that regulates the response of ISCs to oral infection. Regrettably, no tests are available to identify the roles of hemocyte-expressed signals in other intestinal actions. While significant progress has been made in establishing the potential mechanisms by which hemocytes regulate intestinal regeneration, in-depth analyses employing hemocytes are required to define correlations with other aspects of biological processes, such as intestinal physiology and metabolic processes.

#### Fat body

Study of signaling pathways involved in the fat body-gut communication model is a fruitful research direction. A recent expansion of the literature has focused on gut-mediated metabolic homeostasis in the fat body. For example, according to Perrimon N and co-workers, the GI tract contains EEs, which secrete gut prohormones related to systemic lipid storage and metabolism (Song et al., 2014). Flies with loss of tachykinin (TK) EEs display an increased lipid level in the fat body. Starvation-induced excess gut TK levels contribute to the loss of systemic lipid storage due to protein kinase A (PKA)-mediated Sterol regulatory element-binding protein (SREBP) inhibition (Song et al., 2014). Additionally, Li et al. have characterized the function of neurotensin (NT) in the modulation of lipogenesis in the fat body (Li J. et al., 2016). An EE-expressed high NT level triggers increased lipid accumulation, which is accompanied by decreased AMP-activated protein kinase (AMPK) activation (Li J. et al., 2016). It will not be long before other EE-expressed signals are discovered, thus expanding the repertoire of links between the intestinal endocrine system and systemic lipid metabolism. Furthermore, these studies raise another interesting question: Are EEs the only cell type associated with systemic lipid accumulation? An area of obvious interest might be the relationship between EC-mediated nutrient uptake and systemic homeostasis. Remarkably, a key trigger of lipid accumulation appears to be intestinal acidity, which was confirmed by an interesting investigation of an obese-like phenotype suggesting that the level of lipid accumulation is down-regulated in flies with intestinal acid suppression (Lin et al., 2015). Jasper H and co-workers have addressed the complexity of several gut-expressed stress-protective genes with respect to metabolic homeostasis (Biteau et al., 2010). The overexpression of heatshock protein 68 (hsp68) and jafrac1 in intestinal precursors results in the suppression of metabolic decay. The importance of gut-expressed signals in energy metabolism is particularly highlighted by two interesting studies. First, a recent finding identified a role of PGC-1 from the intestine in controlling this metabolic process (Rera et al., 2011). PGC-1 overexpression enhances mitochondrial activity. Importantly, this causes significant increases in both the amount of stored glycogen and free glucose levels, accompanied by a decrease in triglyceride (TAG) levels, supporting the role of PGC-1 in maintaining energy homeostasis (Rera et al., 2011). Second, according to Kohyama-Koganeya et al. systemic energy homeostasis also depends on Bride of sevenless (BOSS) activity from neurons in the brain and EEs in the intestine (Kohyama-Koganeya et al., 2015). Additionally, systemic antimicrobial peptides (AMPs) are also affected by the intestinal immune response. The fat body secretes AMPs to directly remove ingested pathogens. The levels of AMP expression in the fat body are associated with the gut-expressed PGRP-LE, which can be suppressed by several gut-expressed amidase peptidoglycan-recognition proteins (PGRPs), such as PGRP-LB and PGRP-SCs (Paredes et al., 2011; Bosco-Drayon et al., 2012). Thus, we suggest that the gut-fat body communication strategy controls and regulates systemic AMP production, which, identical to AMPs from the intestine, also contributes to defense against ingested pathogenic microorganisms (especially pathogens that are capable of crossing the intestinal epithelium).

Several groups have shown another critical strategy by which the fat body signals to the intestine, providing a potential link between fat body-expressed factors and intestinal actions. For example, Zheng and co-workers have demonstrated the involvement of lamin-B from fat body cells in gut hyperplasia during aging (Chen et al., 2014). Fat body-specific lamin-B loss exacerbates immunosenescence, which contributes to the deregulation of the immune deficiency (Imd) signaling pathway in the intestinal epithelium, causing turnover loss (Chen et al., 2014). Interestingly, the nutrient-sensing pathways regulated by the fat body are indispensable for intestinal digestion and absorption. Carbohydrase and lipase levels within the midgut are regulated by Dawdle (Daw) secreted from the fat body, which activates its receptors Baboon^c^ (Babo^c^) and Punt (Put) to reduce carbohydrase and lipase expression by enhancing the Smad2 level (Chng et al., 2014). Taken together, this series of remarkable results suggests the roles of signals from the fat body in controlling multiple aspects of intestinal actions, including aging-related intestinal inflammation and metabolic homeostasis.

#### Brain

In the past few years, brain-to-gut communication models have been an area of intense investigation. Indeed, the brain has been described as the canonical organ for the production of Dilps, and it is now widely acknowledged that the brain directly affects intestinal immunity, physiology and metabolism (Nässel et al., 2013). Recently, several studies sought evidence of links between IPC-Dilps and gastrointestinal homeostasis. For example, it was shown that brain-expressed Dilp2 is essential for ISC self-renewal after damage (Amcheslavsky et al., 2009). Loss of brain-expressed Dilp2 caused an increased number of Phosphorylated-histone 3^+^ (PH3^+^) cells under inflammation, and the damage-induced epithelial layer of flies with systemic Dilp2 loss also became disorganized (Amcheslavsky et al., 2009). However, the molecular architecture underlying this Dilp-mediated intestinal regeneration is not well understood. Interestingly, results obtained by Shen et al. have shown that cAMP response element-binding protein (CREB) and cAMP-regulated transcription coactivator (Crtc) from the brain are indispensable for intestinal energy homeostasis (Shen et al., 2016). The loss of short neuropeptide F (sNPF) in ECs induced by CREB and Crtc suppression results in the deregulation of epithelial integrity, increased levels of AMPs and energy balance defect (Shen et al., 2016). An in-depth understanding of the role of traumatic brain injury (TBI) in intestinal barrier function has been confirmed by observations of increased bacteria, glucose and innate immune response levels following traumatic injury-mediated death (Katzenberger et al., 2015). A recent study has provided important insights into the roles of brain-expressed hormones in intestinal metabolism, suggesting that the level of nutritive sugar-mediated Diuretic hormone 44 (Dh44) in the brain influences intestinal ingestion and digestion (Dus et al., 2015). Dus et al. have shown that D-glucose-induced Dh44 activity in brain neurons, which is transmitted to R2 gut cells, is responsible for intestinal motility and excretion (Dus et al., 2015). However, many questions remain, including the following: Are any other hormones from the brain responsible for this intestinal action? If this is the case, how are these hormones coordinated to adapt normal intestinal metabolic levels to organ demands? Additionally, attempts have been made to analyze the neuropeptide-mediated meal size. Leucokinin receptor (Lkr) neurons in the brain innervate the foregut, and brain neuron-expressed leucokinin neuropeptide (Leuc) and Lkr regulate total food intake (Al-Anzi et al., 2010). A role of AMPK in autophagy has been detected in the brain and gut by Ulgherait et al. who showed that AMPK overexpression in the brain triggered autophagy and slowed intestinal aging (Ulgherait et al., 2014). This study also suggested that brain-expressed *Autophagy-specific gene 1* (*Atg1*) activity is associated with the maintenance of intestinal homeostasis (Ulgherait et al., 2014). Taken together, these findings indicate that the strategy of brain-gut communication might be highly complex, and an understanding of intestinal actions will require a more integrative view of how these signals from the brain are allocated for different purposes.

It is becoming increasingly apparent that the brain also adjusts its performance based on the response of the intestine to changing nutritional conditions. Sano H has suggested that CCHamide-2 (CCHa2) in the intestine is clearly important for this process (Sano, 2015). Nutrient-mediated CCHa2 controls Dilp production in the brain by stimulating the level of its receptor, CCHa2-R, supporting a molecular basis of the gut-brain axis for Dilp levels (Sano, 2015). A separate study has suggested that the level of nutrient restriction-induced Limostatin (Lst) from gut-associated endocrine corpora cardiaca (CC) cells, along with its receptor CG9918, is also involved in Dilp secretion in IPCs (Alfa et al., 2015). In addition to a direct influence on the production of Dilps, other interesting aspects of gut-expressed signals have been documented. For example, according to Ulgherait et al., autophagy in the brain is regulated by gut-expressed AMPK activation (Ulgherait et al., 2014). In summary, the reason for and consequence of this correlation between gut-expressed signals and Dilp production from the brain remain enigmatic. Given that Dilp levels are strongly associated with metabolism, stress responses and lifespan, research into the effects of signals from the GI tract on Dilp levels has advanced our understanding of how the GI tract communicates with the brain to regulate organismal health.

#### Enteric neurons

Only a few years ago, little was known with respect to enteric neuron-gut communication. However, in recent years, progress has been made in this area such that there is now overwhelming evidence suggesting that enteric neurons exert far-reaching effects on GI tract behavior (Cognigni et al., 2011). A direct role of enteric neurons in intestinal physiological homeostasis was confirmed by an interesting study in which neuronal (Hedgehog) Hh loss triggered a defect in janus kinase/signal transducer and activator of transcription (JAK-STAT) signaling pathway levels, leading to the deregulation of EC fate determination (Han et al., 2015). However, the exact mechanisms involved in neuronal Hh-mediated intestinal homeostasis have not been assessed to date. Future experiments should focus on the question of how neuronal Hh interacts with the JAK-STAT signaling pathway to regulate EC differentiation. Recent studies investigating the gut-to-enteric neuron communication model have yet to unravel the roles of enteric neurons in multiple physiological functions. Growing evidence gleaned by Olds WH and Xu T has suggested that the loss of PPK1 in posterior enteric neurons reduces food intake levels through mechanosensory ion channels (Olds and Xu, 2014). According to Kenmoku et al., the maintenance of barrier function is causally linked to a specific subset of enteric neurons innervating the anterior midgut (Kenmoku et al., 2016). The peritrophic matrix and epithelial barrier of flies with loss of activity of these enteric neurons display high permeability (Kenmoku et al., 2016). Taken together, these findings suggest that this neuron-gut communication strategy has far-reaching effects on the regulation of intestinal regeneration and physiology. However, many questions about the link between the intestine and enteric neurons remain. For example, can the intestine signal to enteric neurons? If so, how do gut-expressed factors regulate the enteric nervous system?

#### Visceral muscle (VM)

Research investigating the links between VM and the intestine has clearly progressed at an impressive rate over the last few years. The primary concern of studies investigating the molecular mechanisms regarding VM-to-gut communication is to validate the roles of VM-expressed messages in ISC activity under physiological homeostasis and under stress conditions. In this section, we will focus on depicting a high-resolution picture of mitogenic signals from VM. According to Xi R and co-workers, VM acts as an ISC niche to secrete signals affecting ISC activity and intestinal homeostasis (Lin et al., 2008). Loss of wingless (wg) in VM reduces the Notch level in the intestine, causing defects in ISC proliferation and differentiation; on the contrary, flies with wg overexpression in VM display excessive ISC-like cells (Lin et al., 2008). Agaisse H and co-workers investigated the role of the VM-expressed JAK-STAT signaling pathway in the stimulation of ISC division in response to challenge (Zhou et al., 2013). Infection-induced Upd3 in ECs activates the JAK-STAT signaling pathway in VM, which is responsible for ISC division by secreting the ligand Vn (Zhou et al., 2013). Additionally, similar to the influence of hemocyte-expressed Dpp on intestinal regeneration after infection, Dpp has been shown to be expressed in VM using a Dpp-LacZ line, maintaining bone morphogenetic protein (BMP) signaling in the midgut and regulating intestinal homeostasis (Guo et al., 2013; Zhou et al., 2015). In addition to VM, ECs act as a niche to control homeostasis and damage-induced regeneration. For example, several studies have revealed a key role of EC-expressed Dpp in lineage differentiation, especially copper cell differentiation, under physiological homeostasis (Li H. et al., 2013; Tian and Jiang, 2014). An important link between diet and Dilp production in VM was established by Amcheslavsky et al., who suggested that nutrient-stimulated TK activity in EEs is associated with VM-specific Dilp3 secretion, contributing to ISC division and intestinal growth in newly eclosed flies (Amcheslavsky et al., 2014). The role of VM-specific signals in ISC proliferation through EEs has been revealed by another study. Paracrine Bursicon (Burs) from EEs with its receptor *Drosophila* leucine-rich repeat-containing G protein-coupled receptor 2 (DLGR2) reduces the Vein (Vn) level by stimulating cyclic AMP (cAMP) activation in VM, which influences ISC proliferative activity and intestinal homeostasis (Scopelliti et al., 2014). Although the major focus of research on VM-gut communication models has been in the context of the response of ISC proliferative activity to VM-expressed signals, there is now a growing emphasis on studying VM-mediated altered modes of ISC division. The contribution of a shift in stem cell behavior in tissue remodeling has been reported by Bilder D and co-workers. VM-specific Dilp3 and systemic Dilp2, 5 drive intestinal growth through ISC symmetric division in freshly eclosed flies after food ingestion (O'Brien et al., 2011). Taken together, these findings suggest that despite significant evidence supporting the mechanical regulation of ISC actions by VM, the next big challenge is how the signals secreted from VM affect intestinal physiological and metabolic homeostasis.

#### Intestinal trachea, ovary, body muscle, and MT

It is becoming increasingly clear that the recognition of signals from the intestinal trachea may be a strategy that largely controls intestinal regeneration and homeostasis. A remarkable discovery in the gut-to-trachea communication model by Lin X and co-workers has suggested that trachea-expressed Dpp is highly correlated with midgut homeostasis, underscoring the importance of roles of the intestinal trachea in ISC proliferation (Li Z. et al., 2013). However, the role of trachea-expressed Dpp in intestinal homeostasis and regeneration is extremely controversial. Indeed, Dpp expression appeared in the intestinal trachea of unchallenged flies and was strongly induced in the intestinal trachea after injury (Guo et al., 2013; Li Z. et al., 2013). Recent data have suggested that the loss of trachea-expressed Dpp has no effect on impaired BMP levels in ISCs and their proliferative activity after intestinal damage (Guo et al., 2013). Why does this phenomenon occur? There are at least two possibilities. (1) The simplest explanation would be that injury triggers the extensive activation of multiple signaling pathways (such as the epidermal growth factor receptor (EGFR) and JAK-STAT pathways) that can sufficiently regenerate the intestinal epithelium, but trachea-expressed Dpp is a secondary mitogenic signal at this moment. (2) We do not know how Dpp is transmitted from the trachea to the intestine. Thus, despite an increased Dpp level after the ingestion of reagents (such as paraquat), we speculate that injury also causes serious defects in tracheal activity, which might contribute to preventing the transformation of the trachea to the intestine. The nature of the signaling pathways responsible for this trachea-gut communication model is still unknown, and an understanding of how trachea-expressed Dpp is integrated into the intestine under homeostasis and inflammation are among future challenges. It is anticipated that the influence of trachea-expressed signals on intestinal actions will be manifold (they might contribute to intestinal physiology and metabolism) and is only starting to be understood. Furthermore, the roles of gut-expressed signals in tracheal actions remain enigmatic. In consideration of the aforementioned notion that Dpp from VM and EC plays an important role in intestinal regeneration, these models raise two additional important questions regarding why the same factor from different niches (such as the VM, intestinal trachea and ECs) regulates similar biological processes and how an ISC senses specific Dpp signals from different niches to affect intestinal homeostasis.

Next, this review will focus on the gut-ovary/body muscle axes. Recently, Perrimon and co-workers has shown that systemic organ wasting is related to the level of ISC proliferation. ImpL2 secreted from the intestine with yorkie (Yki) loss reduces systemic insulin/insulin-like growth factor (IGF) signaling, establishing ovary, muscle and fat body wasting (Kwon et al., 2015). A role of juvenile hormone (JH) signaling has been proposed in the regulation of mating-mediated intestinal remodeling. Intestinal remodeling in females is regulated by the JH, Methoprene-tolerant (Met) and Germ cell-expressed (Gce) receptors after mating, which drives reproductive remodeling (Reiff et al., 2015). Additionally, it should be noted that intestinal AMPK activation regulates muscle homeostasis during aging (Ulgherait et al., 2014). Taken together, these findings support the notion that gut-expressed signals appear to be responsible for body muscle and ovary behaviors.

**Figure 2:**
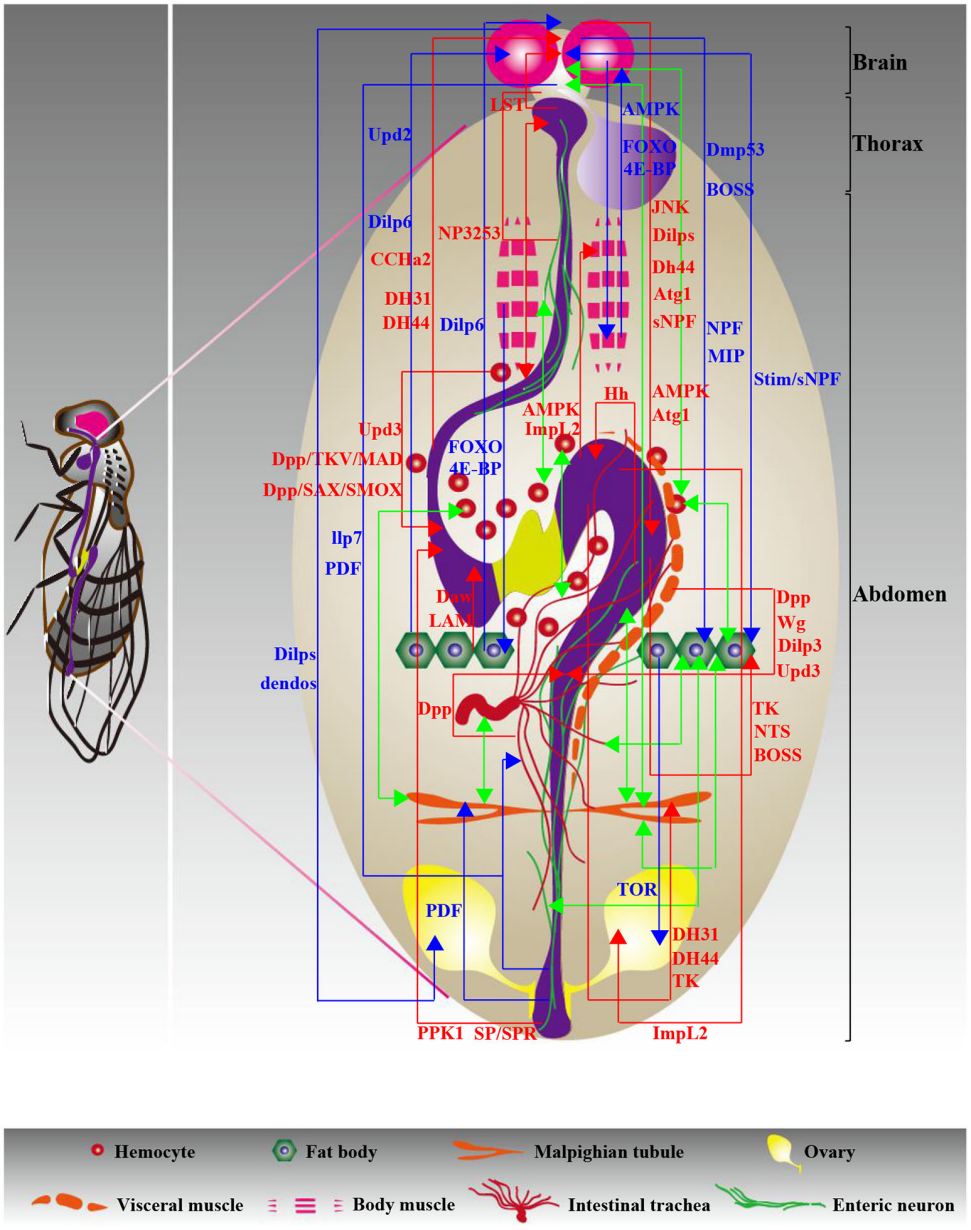
**Diagram of interorgan communication in adults**. The multifaceted molecular foundations underlying communication play a central role in the peaceful coexistence of organs, contributing to the maintenance of organismal health. Different types of organ-organ communication axes are color coded as follows: red and blue indicate gut-neighbor axes and neighbor-neighbor axes, respectively, and green indicates unknown axes. The relevant signals are exhibited and detailed descriptions are shown in Tables 1, 3.

Intriguingly, the basic biological processes of the renal system are also regulated by intestinal behaviors. The observation that gut-expressed signals cooperate with their receptors within the MTs to affect renal function was made by Talsma et al., who revealed the myotropic effects of pigment-dispersing factor (PDF), a central neuropeptide PDF (Talsma et al., 2012). Intestinal PDF activates its receptor, PDF receptor (Pdf-R), which is expressed by muscles in the MTs to control ureter contractions by enhancing cAMP activity (Talsma et al., 2012). Additionally, Nässel DR and co-workers presented evidence that gut-expressed TK, as a circulating hormone, triggers Dilp5 activation in the principal cells from MTs through the TK receptor (TK-R) to affect stress resistance (Söderberg et al., 2011). This finding was confirmed by the observation of increased survival under stress of TK-R, Dilp5 and *Drosophila* insulin receptor (dInR) mutants (Söderberg et al., 2011). Although existing data indicate that gut-expressed signals are important regulators of MT behaviors, it remains largely unknown whether these signals coordinate MT-expressed signals with the emergence of nephropathy. A summary of the information relevant to gut-neighbor axes in adults is presented in Figure 2 and Table 1. To date, most of our knowledge about peripheral organs associated with adult intestinal actions comes from the brain and fat body. Many points need to be further clarified to understand the intestinal actions caused by signaling pathways from other peripheral organs, including the intestinal trachea and enteric neurons.

**Table 1:** **Summary of the gut-neighbor axes in adults**.

**Neighbors**	**Signals**	**Comments**	**Sources**
He	JNK, Upd3, JAK-STAT	Intestinal renewal	Chakrabarti et al., 2016
He	Dpp/TKV/MAD, SAX/SMOX	Intestinal renewal	Ayyaz et al., 2015
IT	Dpp	Intestinal renewal	Li Z. et al., 2013
FB	TK, PKA/SREBP	Physiology, metabolism	Song et al., 2014
FB	Boss	Physiology, metabolism	Kohyama-Koganeya et al., 2015
FB	PGRP-LE	Immune response	Bosco-Drayon et al., 2012
FB	PGRP-SCs, PGRP-LB	Immune response	Paredes et al., 2011
FB	lamin-B, Imd pathway	Intestinal renewal	Chen et al., 2014
FB	Daw, Babo^c^, Punt, TGF-b/Activin	Physiology, metabolism	Chng et al., 2014
FB	NT	Physiology, metabolism	Li J. et al., 2016
EN	Hh, JAK-STAT	Intestinal renewal	Han et al., 2015
EN	ovo, SP, Leucokinin, Dilp2, Dilp7	Physiology, metabolism	Cognigni et al., 2011
EN	NP3253	Intestinal structure	Kenmoku et al., 2016
EN	PPK1	Absorption, digestion	Olds and Xu, 2014
EN	PDF, PdfR, cAMP	Excretion	Talsma et al., 2012
Br	Dilp2, InR	Intestinal renewal	Amcheslavsky et al., 2009
Br	Crtc, CREB, sNPF	Immune response, stress resistance	Shen et al., 2016
Br	*AttC, DiptB, Mtk*	Intestinal permeability	Katzenberger et al., 2015
Br	Dh44	Absorption, digestion, excretion	Dus et al., 2015
Br	CCHa2, CCHa2-R, Dilps	Physiology	Sano, 2015
Br	AMPK, Atg1	Intestinal autophagy, homeostasis.	Ulgherait et al., 2014
Br	Lst, Dilps	Metabolism	Alfa et al., 2015
Br	Leuc, Lkr	Food intake	Al-Anzi et al., 2010
Br	JNK, Dilp2	Stress response	Karpac et al., 2009
BM	AMPK	Muscle homeostasis	Ulgherait et al., 2014
Ov, FB, BM	Yki, Imp-L2, Insulin/IGF	Tissue wasting	Kwon et al., 2015
VM	Dpp	Intestinal renewal	Guo et al., 2013; Zhou et al., 2015
VM	Wg	Intestinal renewal	Lin et al., 2008
VM	TK, Dilp3	Intestinal renewal	Amcheslavsky et al., 2014
VM	Bur, DLGR2, cAMP, Vn	Intestinal renewal	Scopelliti et al., 2014
VM	Dilp3	Intestinal renewal	O'Brien et al., 2011
VM	Upd3, JAK-STAT, Vn/EGFR	Intestinal renewal	Zhou et al., 2013
MT	TK, TKR, Dilp5, InR	Stress resistance	Söderberg et al., 2011
MT	PDF, PdfR, cAMP	Renal function	Talsma et al., 2012

*He, hemocyte; IT, intestinal trachea; FB, fat body; EN, enteric neuron; Br, brain; BM, body muscle; Ov, ovary; VM, visceral muscle; MT, malpighian tubule*.

## Neighbor-neighbor axes

Attempts to underscore the relationships between peripheral organs are currently underway. In this section, we review emerging data supporting the existence of neighbor-neighbor axes in adult *Drosophila*. The influences of these neighbor-neighbor communication strategies on different biological aspects will be discussed in detail.

### Reproductive behaviors

Research into how precise mechanisms regulate stem cell activity has been most extensively conducted in reproductive systems, yet much is still unknown regarding how signals from other organs achieve this process. The greatest progress in our knowledge of the extrinsic factors regulating reproductive behavior has come from studies of Dilps from the brain, which are strongly associated with germline stem cell (GSC) proliferation. Ikeya et al. established a causal link between female GSC proliferative activity and brain-expressed Dilp levels. Defects in brain-expressed Dilp activity are strongly associated with the loss of egg production (Ikeya et al., 2002). In support of this model, Liu et al. suggested that ovarian maturation in the first days of adult life can be regulated by brain-expressed Dilp1 (Liu et al., 2016). Additionally, a previous study reported that IPC-expressed Dilps control GSC division and vitellogenesis (LaFever and Drummond-Barbosa, 2005). Flies with IPC loss have severely impaired follicle cell proliferation ability in response to nutritional input. Several factors cooperate with IPC-expressed Dilps to affect the reproductive system. For example, a recent study implicated a role of brain-produced *Drosophila* alpha-endosulfine (dendos) protein in normal rates of egg chamber development and follicle cell proliferation by regulation of Dilp levels (Drummond-Barbosa and Spradling, 2004). Dendos loss in the brain causes a defect in the proliferative response of ovarian cells to nutritional changes (Drummond-Barbosa and Spradling, 2004). A fat body-ovary axis with respect to GSC status has been reported. Target of Rapamycin (TOR) activation in the fat body is triggered by high amino acid levels, which plays an important role in GSC maintenance and ovulation rates; however, lower amino acid levels cause increased GCN2 kinase activity in the fat body, leading to GSC loss (Armstrong et al., 2014). In summary, reproductive behaviors thus seem to be caused by a large number of systemic changes that influence GSC maintenance and self-renewal.

### Stress resistance and lifespan

There is growing recognition that brain-to-fat body communication is involved in stress resistance and lifespan. First, we provide several interesting findings with respect to the roles of several communication strategies stress responses. For example, Bai et al. suggested that the Dilp6 level in the fat body is deeply involved in the regulation of brain-expressed Dilp2, 5 (Bai et al., 2012). Fat body-expressed Dilp6 overexpression increases the level of transcriptional target of dFOXO, which triggers decreased activity of hemolymph Dilp2 and brain-expressed Dilp2, 5, contributing to improving oxidative stress resistance and extending lifespan (Bai et al., 2012). According to Bauer et al., the reduced Dilp2 and insulin signaling levels in the fat body are thought to be mediated through the dysfunction of IPC-expressed *Drosophila melanogaster* p53 (Dmp53), accompanied by lifespan extension (Bauer et al., 2007). The key role of IPC-expressed jun-N-terminal kinase (JNK) activity in an organism's stress response was confirmed by a recent report showing that the repression of Dilp2 triggered by IPC-expressed JNK activation resulted in reduced insulin signaling levels in the periphery, such as the fat body, which is responsible for increasing stress tolerance (Karpac et al., 2009). Taken together, these findings show that brain-to-fat body communication strategy is crucial for stress responses, and deregulation of this strategy has important deleterious consequences for stress resistance.

Next, the alteration of lifespan appears to involve an imbalance of communication between organs. According to Demontis F and Perrimon N, muscle-expressed FOXO, and 4E-BP activity mitigates the loss of proteostasis in the aging retina, brain and fat body (Demontis and Perrimon, 2010). FOXO and 4E-BP achieve these outcomes by triggering insulin release and stimulating 4E-BP activity in other tissues (Demontis and Perrimon, 2010). One finding on AMPK activity was that AMPK overexpression in the brain induces autophagy, which is responsible for slowing muscle aging and prolonging lifespan (Ulgherait et al., 2014). Additionally, injury-induced Upd3 activity in hemocytes by the JNK signaling pathway stimulates the level of JAK-STAT signaling in the fat body to promote survival of flies, suggestive of a hemocyte-to-fat body communication model (Chakrabarti et al., 2016). The exact mechanisms that determine the organ-organ communication associated with lifespan are not yet known, but understanding this is evidently key to learning precisely how lifespan is regulated, at least in the adult *Drosophila* model.

### Metabolic homeostasis

Several studies have further explored organ-organ communication models associated with metabolic homeostasis. For example, a recent study implied a role of fat body-expressed Dilp6 in starvation tolerance. Fat body-expressed Dilp6 stimulates insulin-signaling activity in oenocytes, which is critical for lipid turnover in response to fasting (Chatterjee et al., 2014). It was shown that feed-mediated Upd2 production in the fat body activates the JAK-STAT signaling pathway from GABAergic neurons innervated in IPCs to trigger the release of Dilps in the brain, which have the capacity to control energy balance and systemic growth (Rajan and Perrimon, 2012). Interestingly, considering the role of fat body-brain communication strategies in improving oxidative stress resistance and extending lifespan described above, we suggest that the same organ-organ communication model might be associated with different aspects of biological processes. Taken together, these findings suggest that the cues associated with the brain-fat body axis might be more than just a means of understanding how these two organs collaborate to regulate metabolic homeostasis. This raises the real possibility that a dysfunctional interorgan communication network might contribute to the emergence of metabolic diseases. A summary of the neighbor-neighbor axes in adults is provided in Figure 2 and **Table 3**.

## Links between larval organs

Thus far, we have discussed data substantiating the notion that dialogs between organs are essential processes in the life of adult *Drosophila*, involving the maintenance and regulation of homeostasis, reproductive behavior, stress resistance, lifespan, and other process. Although our understanding of organismal development and growth is most highly advanced in larvae, there is still much to be learned about interorgan communication at this developmental stage. In this part of the review, we focus specifically on depicting a high-resolution picture of organ-to-organ communication models in larvae.

### Gut-neighbor communication models

#### Fat body and prothoracic gland

We first emphasize the functional contribution of the gut-fat body axis. Hemocytes act as effective mediators that are pivotal in the communication between gut-expressed ROS/nitric oxide (NO) and fat body-expressed AMP production. The Diptericin (Dpt) level in the fat body is affected by the intestinal NO activity triggered by ROS (Wu et al., 2012). Glittenberg et al. presented evidence of the ability of pathogen and gut signals to communicate with the fat body, leading to the activation of systemic Toll-dependent immunity (Glittenberg et al., 2011). Candida-produced secreted aspartyl protease 4 (SAP4) and SAP6 and gut-produced NO contribute to Drosomycin (Drs) secretion from the fat body (Glittenberg et al., 2011). These findings suggest that a consequence of the loss of gut-expressed signals is a defect in systemic immune responses, yet the exact mechanisms responsible for gut-mediated systemic immune defects have yet to be investigated. Additionally, one recent study implicated Gp93 as an effective regulator of intestinal function and lipid catabolism (Maynard et al., 2010). Flies missing Gp93 display several defects in the midgut, accompanied by insulin loss and greatly enhanced fat body lipid catabolism (Maynard et al., 2010).

Of particular interest is the prothoracic gland, which also adjusts its performance based on the signals from gut. To date, two studies have analyzed the intestinal actions that mediate signaling levels in the prothoracic gland. Hh appears to be a gut-expressed factor that possesses the ability to couple nutrition to growth and developmental progression (Rodenfels et al., 2014). Tissue growth is regulated by circulating Hh that signals to the fat body. Ecdysteroid production in the prothoracic gland is also controlled by gut-expressed Hh, contributing to developmental timing (Rodenfels et al., 2014). Additionally, modulatory control of prothoracic gland behavior also occurs through the actions of intestinal symbiotic bacteria. Research investigating the role of *Lactobacillus plantarum*, an intestinal commensal bacterium, in systemic growth has shown that gut-resident *Lactobacillus plantarum* (*L. plantarum*) triggers TOR activation in the fat body and prothoracic gland upon nutrient scarcity, which contributes to optimal development through Dilps and ecdysone secretion from the brain and prothoracic gland, respectively, supporting an interesting link between this intestinal commensal bacterium and hormonal growth signal production (Storelli et al., 2011). Therefore, the gut-prothoracic gland communication strategy is thought to be a novel contributor to the regulation of larval development. Increased emphasis is also being placed on the role of corpora allata-prothoracic gland communication strategy in development, which will be discussed below. However, how the dialog between the prothoracic gland and other organs is established remains an intriguing question.

#### Brain, MT, and imaginal discs

Research on signaling crosstalk between the brain and intestine has seen impressive advances in the past few years. A novel signal CCHa2, which is discussed above for its role in regulating Dilp levels in adult brain, has also been found to be an important component of brain–gut signaling pathway in larvae. For example, Li et al. reported the exciting observation that the brain receives feed-induced CCHa2 from gut endocrine cells through its receptor (Li S. et al., 2013). Furthermore, another study by Ren et al. has shown that food intake is regulated by CCHa2 in larval brain (Ren et al., 2015). Because flies with a dysfunction in CCHa2 show a significantly reduced food intake and have a remarkably delayed development (Ren et al., 2015). They also found that these CCHa2 inactivation-induced defects might be due to the decreased Dilp2, 3 levels (Ren et al., 2015). Thus, these studies suggest that CCHa2 acts as a key signal in brain to be clearly critical for regulation of gut nutrient uptake and developmental timing. Interestingly, brain–gut axis is not only essential for food intake but also important strategy that control intestinal motility. For example, four serotonergic neurons in the brain have been characterized by Schoofs et al., who suggested that central serotonergic neurons, as a conduit, play an important role in movements of the esophagus and proventriculus, establishing a brain–gut neural pathway involved in foregut motility (Schoofs et al., 2014). We suggest that, like CCHa2, these neurons could be implicated in regulation of intestinal absorption. Taken together, these studies illustrate how the brain–gut communication strategy impinges on larval food intake.

Gut-expressed factors also regulate the actions of other peripheral organs, such as the MTs and imaginal discs. A tantalizing study has shown that, in both larvae and adults, the Diuretic hormone 31 receptor (DH31-R) and Diuretic hormone 44 receptor (DH44-R) signaling pathways in neurons of the central nervous system (CNS) and MTs are activated by the gut peptides DH31 and DH44 by triggering cAMP activation (Johnson et al., 2005). Furthermore, the receptor component protein (RCP) is utilized by DH31-R (Johnson et al., 2005). One issue remains, however—the further outcome of gut-expressed hormones on MT actions. Additionally, the field of interorgan communication in larvae is currently attracting renewed interest, especially concerning the influence of gut-expressed signals on the wing imaginal disc. For example, a recent study investigating organ lipid transport has shown that the neutral and polar lipid composition of the brain and wing imaginal disc is controlled by the mobilization of gut-produced lipid through Lipid Transfer Particle (LTP) and lipoprotein Lipophorin (Lpp) activation in the fat body (Palm et al., 2012). This result suggest that there is a lipid transport channel linking intestinal lipid levels and lipid composition of the brain and wing imaginal disc in larvae.

#### Enteric neurons and VM

**Figure 3:**
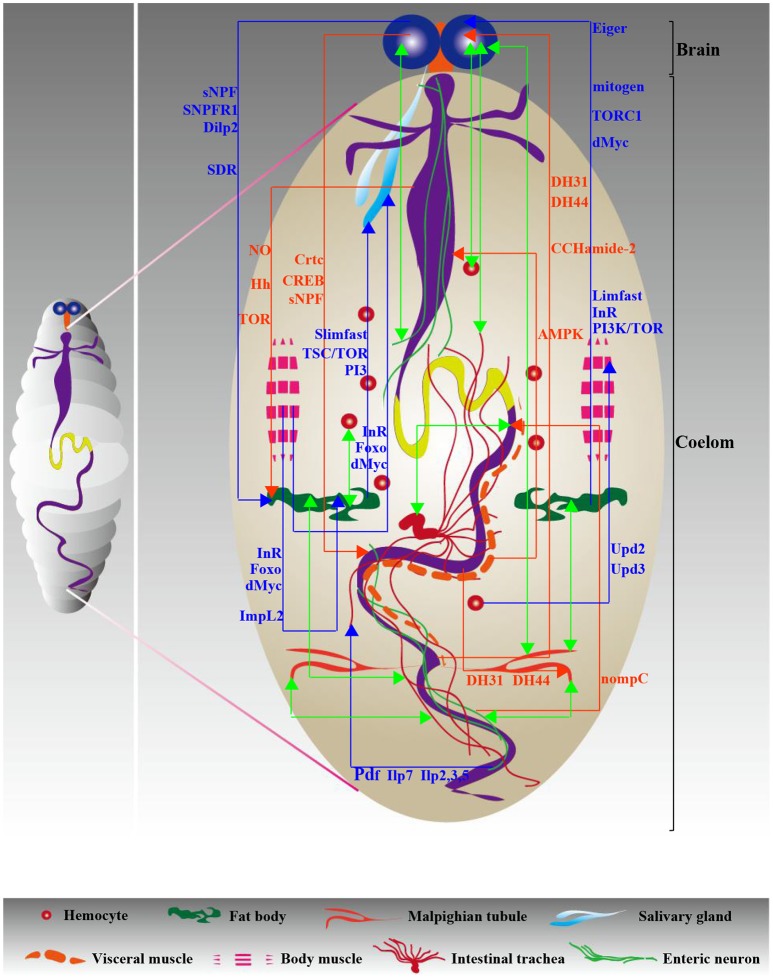
**Diagram of interorgan communication in larvae**. Similar to adults, the signaling network necessary for interorgan communication ensures a steady state in the larval body cavity. Red and blue indicate gut-neighbor axes and neighbor-neighbor axes, respectively; green indicates unknown axes. The relevant signals are exhibited and detailed descriptions are shown in Tables 2, 3.

A novel cell type that communicates with the GI tract in larvae was recently revealed: the enteric neurons. A fascinating study investigating the regulation of defecation behavior has suggested a link between enteric neurons and intestinal muscle (Zhang et al., 2014). The motor neurons innervating the hindgut and anal sphincter cooperate with the intestinal muscle to control defecation activity (Zhang et al., 2014). Although striking advances have been made in recent years, the exact regulatory network of links between enteric neurons and the intestine is only partly understood. This is probably due to the complexity of the signaling pathways responsible for this communication model. Additionally, Bland et al. presented data revealing the regulation of peristalsis through AMPK activity from VM, contributing to intestinal function and organismal growth (Bland et al., 2010). However, contrary to the abundant contributions of VM-gut communication in adults, functional impacts of this communication strategy in larvae are still poorly understood. Evidence uncovering the other signals from VM that regulate intestinal function should be available in the near future. Details of gut-neighbor communication models in larvae are summarized in Figure 3 and Table 2. This review next summarizes numerous studies that have investigated well-established non-gastrointestinal organ communication models in larvae acting as dedicated strategies regulating multiple aspects of tissue growth, stem cell actions, development and other biological processes.

**Table 2:** **Summary of the gut-neighbor axes in larvae**.

**Neighbors**	**Signals**	**Comments**	**Sources**
He, FB	NO, *Dpt*	Immune response	Wu et al., 2012
EN	nompC	Defecation	Zhang et al., 2014
Br	CCHa2	Metabolism	Li S. et al., 2013
Br	CCHa2	Food intake	Ren et al., 2015
Br	–	Intestinal motility	Schoofs et al., 2014
VM	AMPK	Peristalsis, organismal growth	Bland et al., 2010
Br, MT	DH31, DH44	Neural, renal function	Johnson et al., 2005
FB, Br, WID	LTP, Lpp	Metabolism	Palm et al., 2012
FB, PG	Hh	Development, organismal growth	Rodenfels et al., 2014
FB, Br, PG	TOR, Dilps, ecdysone	Organismal growth	Storelli et al., 2011
FB	NO	Immune response	Glittenberg et al., 2011
FB	Gp93	Metabolism	Maynard et al., 2010

*He, hemocyte; FB, fat body; EN, enteric neuron; Br, brain; VM, visceral muscle; MT, malpighian tubule; WID, wing imaginal disc; PG, prothoracic gland*.

### Non-gastrointestinal organ communication axes

#### Tissue growth

The influence of interorgan communication axes on tissue growth has long been of interest and is being actively investigated in larvae. For example, growing evidence gleaned from several studies has suggested the regulation of tissue growth by the brain-fat body axis. We outline the current data on this communication model below. First, the putative involvement of brain-expressed Dilps and fat body-expressed insulin in larval tissue growth has been evaluated by several interesting reports. For example, Lee et al. showed that sNPF and sNPF receptor 1 (sNPFR1) stimulate Dilp2 activity through the regulation of extracellular signal-related kinases (ERK) in IPCs (Lee et al., 2008). Secreted Dilp2 acts as a key regulator to affect insulin signaling levels in the fat body, which is strongly related to growth and metabolism. Furthermore, the influence of fat body-expressed *Drosophila* Myc (dMyc) on organismal growth has been shown by Parisi et al., who suggested that dMyc activity from the fat body is responsible for the release of Dilp2 from the brain, contributing to the systemic increase in size (Parisi et al., 2013). In an interesting model presented by Okamoto et al., glia-expressed secreted decoy of InR (SDR) binding to circulating Dilps affects insulin/IGF signaling activity in peripheral organs, contributing to organismal growth (Okamoto et al., 2013). The faster rate is triggered by SDR loss-induced increased insulin levels, thereby leading to increased body size. According to Léopold P and co-workers, a humoral signal is released into the hemolymph through TOR signaling pathway in the fat body to stimulate the secretion and accumulation of Dilps in IPCs (Géminard et al., 2009). All of these observations demonstrate that brain-to-fat body communication strategy control larval growth through Dilps and insulin signals. Second, other signals from the brain and fat body are also associated with this biological process. For example, Agrawal et al. argued that TNF-α converting enzyme (TACE)-mediated Eiger (Egr) secretion from the fat body contributes to organismal growth through Grindelwald (Grnd) activity in the brain in response to a normal protein diet (Agrawal et al., 2016).

Additionally, our current knowledge of organ-organ communication models responsible for salivary gland growth is rudimentary. To date, two studies have investigated the well-established regulators that largely control salivary gland growth. A functional role for the InR-Foxo-dMyc signaling pathway in larval skeletal muscles was suggested following the discovery that InR/Foxo-induced dMyc transcriptional activity in muscles affects the growth of muscles and other peripheral tissues, such as the salivary glands and fat body, suggesting a requirement for InR-Foxo-dMyc for the size of skeletal muscles and other tissues (Demontis and Perrimon, 2009). Additionally, a question pertaining to the global growth of fat body control has been solved by Colombani et al. Slimfast (Slif) loss in the fat body causes a dysfunction in tuberous sclerosis complex (TSC)/TOR signaling, leading to the inhibition of salivary gland growth induced by phospho-inositide 3-kinase (PI3K) inactivation (Colombani et al., 2003). The above examples of the regulation of salivary gland growth caused by signals from skeletal muscles and the fat body beg the following questions: What are the consequences of changes in salivary gland growth? In addition to signals from these two organs, can we identify circulating signals from other peripheral organs that control salivary gland behavior?

Several uncommon interorgan communication models have recently been elucidated. As mentioned above, two studies have identified gut-expressed signals as being of critical importance in regulating prothoracic gland actions and contributing to tissue growth (Storelli et al., 2011; Rodenfels et al., 2014). Interestingly, in addition to the gut-prothoracic gland communication strategy, signals from other specific organs are essential for tissue growth by regulating prothoracic gland actions. For example, the insulin-dependent regulation of tissue growth by JH release was demonstrated in the corpora allata (Mirth et al., 2014). Experiments have shown that flies lacking corpora allata-expressed JH have decreased levels of insulin signaling caused by ecdysone synthesis in the prothoracic gland, leading to a defect in body size (Mirth et al., 2014). Additionally, Linneweber et al. functionally linked tracheal cellular growth to enteric neuron activity, providing strong evidence supporting the regulation of terminal cell growth in the posterior tracheal branches by Dilp7- and PDF-Gal4-positive neurons innervated around the hindgut via insulin signaling based on metabolic status (Linneweber et al., 2014). Taken together, these findings suggest that, to maintain healthy regulation of tissue growth, harmonious coordination between organs is required. If this delicate balance fails, tissue growth defects may develop.

#### Stem cell actions

What is the regulatory machinery underlying the interorgan communication that largely controls stem cell actions in larvae? While still a work in progress, our understanding in this field has increased significantly in recent years. In fact, apart from a few analyses, most conclusions related to the interorgan communication responsible for stem cell actions have been formulated based on studies conducted in the larval CNS. For example, the fat body-brain axis, in addition to its role in regulating tissue growth, acts as a dedicated channel to affect stem cell capability in the hematopoietic system. Shim et al. highlighted the role of IPC-expressed Dilp2 in orchestrating hematopoietic progenitor fate, suggestive of the causal link between the brain and lymph gland (Shim et al., 2012). The fat body, which responds to amino acids, stimulates the secretion of Dilp2 from IPCs, which is directly sensed by the InR-dTOR-wg signaling pathway in the medullary zone (Shim et al., 2012). This finding has undoubtedly been instrumental in improving our understanding of how signals from the peripheral organs regulate hemopoiesis in larvae. Additionally, there are several indications that the proliferative capability of neuroblasts (NBs) is also the target of this channel. For example, according to Britton and Edgar, a novel mitogen from the fat body is deeply involved in the proliferative activity of NBs in the larval CNS (Britton and Edgar, 1998). Sousa-Nunes et al. suggested that the fat body-specific activation of Slif, InR and PI3K/TOR causes NBs to exit quiescence (Sousa-Nunes et al., 2011). Dilps from glial cells are required for NB reactivation, supporting a role of fat body-glia-NB relay in proliferation. A link between NBs and glial cells has been reported by another study showing that Dilp secretion from glial cells stimulates PI3K/Akt levels in NBs, which drives NB proliferation (Chell and Brand, 2010). Taken together, most studies of cross-talk-associated stem cell actions to date have been centered on the regulation of neural stem cells in the larval brain. Future studies will focus on exploring the further consequences of cross-talk-mediated alteration of NB proliferative capacity.

#### Development

Understanding how interorgan communication contributes to larval development is a challenging and fundamental question. Recently, the brain and prothoracic gland have been thought to be the two main organs participating in the regulation of development. Data obtained from Sarraf-Zadeh et al. indicated a local function of Imaginal morphogenesis protein-Late 2 (Imp-L2) in developmental timing (Sarraf-Zadeh et al., 2013). Brain ring gland-expressed Imp-L2 regulates developmental timing through insulin-growth-factor-like signaling (IIS) activity in the prothoracic gland. Loss of Imp-L2 accelerates development, but Imp-L2 overexpression delays pupariation (Sarraf-Zadeh et al., 2013). The work of Layalle et al. revealed a function of the TOR signaling pathway in the prothoracic gland in developmental timing (Layalle et al., 2008). The reduced ecdysone level in the prothoracic gland induced by TOR loss causes growth defects (Layalle et al., 2008). In consideration of the aforementioned notion that the intestine signals to the prothoracic gland, contributing to larval development, we speculate that the prothoracic gland might be a pivotal organ that participates in the regulation of larval development. Additionally, Colombani et al. found that brain-expressed DLGR3 affects growth by regulating the Dilp8-induced developmental delay (Colombani et al., 2015). Colombani et al. has also underscored the important relationships between disc-expressed Dilp8 and developmental timing (Colombani et al., 2012). The Dilp8 thus serves as a crucial node in an organ-organ communication signaling network that governs larval development.

#### Other biological processes

The dialog between hemocytes and somatic muscle has been identified as playing an important role in the immune response. For example, Upd2 and Upd3 from circulating hemocytes activate the JAK/STAT signaling in somatic muscles, which is implicated in the immune defense against wasp infection (Yang et al., 2015). Recently, the focus has shifted to the interorgan communication deeply involved in the functional responses of powered organs, particularly the heart. The establishment of the fat body-heart axis has been confirmed by the interesting observation that TOR suppression specifically in the fat body causes the dysregulation of heart structure and function (Birse et al., 2010). However, it remains to be determined how the heart receives the signals that mediate its biological function. Furthermore, it will be interesting to investigate the influences of signals from other organs on heart behavior in the coming years. Additionally, a study by Owusu-Ansah et al. identified that muscle mitohormesis exerts critical functions in promoting longevity (Owusu-Ansah et al., 2013). A striking finding of this study is that the muscle mitohormesis-mediated lifespan extension is regulated by mitochondrial unfolded protein response (UPR^mt^) and by Imp-L2 secretion from muscles, which suppresses insulin signaling in the fat body (Owusu-Ansah et al., 2013). We have summarized all the information relevant to non-gastrointestinal organ communication axes in larvae (presented in Figure 3 and Table 3). In summary, as is evident from the scarcity of molecular data, data on organ-organ communication in larvae remain lacking, with the exception of the brain-fat body communication model, which has witnessed significant development over the last few years. Furthermore, although our understanding of *Drosophila* gut development is quite advanced, how this biological process is regulated by organ-organ communication in larvae remains largely unknown. Thus, it will be of significant interest to research the larval neighbor-gut communication strategies responsible for these gut developmental processes in the coming years.

**Table 3:** **Summary of the neighbor-neighbor axes**.

**Axes**	**Signals**	**Comments**	**Sources**
FB—Br^A^	Upd2, JAK-STAT, Dilps	Organismal growth, metabolism	Rajan and Perrimon, 2012
FB—Ov^A^	TOR, GCN2	Reproduction	Armstrong et al., 2014
He—FB^A^	JNK, Upd3, JAK-STAT	–	Chakrabarti et al., 2016
FB—Br^A^	Dilp2, 5, 6	Lifespan	Bai et al., 2012
Br—FB^A^	Dmp53, Dilp2	Lifespan	Bauer et al., 2007
Br—FB^A^	JNK, Dilp2	Stress response	Karpac et al., 2009
Br—Ov^A^	Dilp1	Reproduction	Liu et al., 2016
Br—Ov^A^	Dilps	Reproduction	LaFever and Drummond-Barbosa, 2005
Br—Ov^A^	dendos	Reproduction	Drummond-Barbosa and Spradling, 2004
Br—BM^A^	AMPK	Muscle homeostasis	Ulgherait et al., 2014
Br—Ov^A^	Dilps	Reproduction	Ikeya et al., 2002
BM—Re, Br, FB^A^	FOXO, 4E-BP, Insulin	Proteostasis	Demontis and Perrimon, 2010
EN—IT^L^	PDF, Dilp7, Dilp2,3,5	Tissue growth	Linneweber et al., 2014
FB—Br^L^	TORC1, Dilps	Metabolism	Géminard et al., 2009
FB—Br^L^	dMyc, Dilp2	Organismal growth	Parisi et al., 2013
FB—Br^L^	mitogen	CNS	Britton and Edgar, 1998
FB—Br^L^	Slif, InR, PI3K/TOR	CNS	Sousa-Nunes et al., 2011
Br—FB^L^	sNPF, SNPFR1, Dilp2	Organismal growth, metabolism	Lee et al., 2008
Br—PO^L^	SDR, Dilps, Insulin/IGF	Tissue growth	Okamoto et al., 2013
FB, Br—LG^L^	Dilp2, InR-dTOR-wg	Hematopoiesis	Shim et al., 2012
FB—SG^L^	Slif, TSC/TOR, PI3	Tissue growth	Colombani et al., 2003
BM—SG, FB^L^	InR, Foxo, dMyc	Tissue growth	Demontis and Perrimon, 2009
Br—PG^L^	Imp-L2, IIS	Development	Sarraf-Zadeh et al., 2013
He—BM^L^	Upd2, Upd3	Immune response	Yang et al., 2015
CA—PG^L^	JH, Insulin, ecdysone	Development, organismal growth	Mirth et al., 2014
FB—Br^L^	Eiger, TACE, Grnd	Organismal growth	Agrawal et al., 2016
FB—Ht^L^	TOR	Heart structure, function	Birse et al., 2010
BM—FB^L^	Imp-L2	Lifespan	Owusu-Ansah et al., 2013

*FB, fat body; Br, brain; Ov, ovary; He, hemocyte; BM, body muscle; Re, retina; IT, intestinal trachea; EN, enteric neuron; PO, peripheral organ; LG, lymph gland; SG, salivary gland; PG, prothoracic gland; CA, corpora allata; Ht, heart; A, adults; L, larvae*.

## Conclusion and perspectives

Studies in the accessible *Drosophila* model have made significant progress in elucidating the functions of organ-organ communication strategies and the consequences of their deregulation for homeostatic equilibrium. However, the key issue regarding how the signaling pathways necessary for this interorgan communication integrate with each other remains incompletely understood. This review merely outlines some of the basic research areas regarding the interactions between *Drosophila* organs and tissues that contribute to the organism's homeostasis and health. Several fascinating topics in this field have yet to be clarified and discussed.

Regenerative inflammatory signals are considered strongly associated with organismal health and diseases (Panayidou and Apidianakis, 2013). Notably, there is growing recognition of the multiple signaling pathways responsible for intestinal regeneration in the adult *Drosophila* intestine, which are not exclusively confined to the intestine itself but also present in the peripheral organs. For example, as discussed earlier, existing data indicate that a stress-induced change in ISC proliferative activity is caused by Dilp2 from the brain (Amcheslavsky et al., 2009). Fundamental questions that have arisen from this preliminary work include the following: Are there other types of Dilps that could also contribute significantly to ISC activity? If so, what are the exact programs underlying excreted Dilp-induced ISC self-renewal? What are the functional differences between these Dilps regarding intestinal inflammation? Furthermore, another challenge will be to determine how all these Dilps might be integrated during this regenerative process. Therefore, understanding the functions of brain-produced Dilps in stem cell biology will undoubtedly lead to a better understanding of the functional roles of the brain-gut communication model in promoting regenerative inflammation and, potentially, cancer. Interestingly, brain-expressed Dilp1, but not Dilp2, was identified as facilitating ovarian maturation in the first days of adult life (Liu et al., 2016). It remains largely unknown why different brain-produced Dilps control stem cell actions in different biological systems. To date, few studies have provided essential clues to answer this question.

Although much progress has been made in our understanding of intestinal inflammation after acute infection, the detailed functions of organ-organ communication in regenerative inflammation during aging represent an additional mystery. A pioneering study conducted by Chen et al. elucidated the impacts of organ-organ communication on aging, intensively suggesting that the age-related decline in the regenerative capacity of ISCs can be affected by age-associated lamin-B loss, specifically in fat body cells during immunosenescence (Chen et al., 2014). In this regard, we speculate that immunosenescence-promoting factors may go beyond the reduction of lamin-B; other abnormal signal levels from the fat body might also be responsible for age-associated intestinal diseases, a notion that requires further investigation. In addition to the fat body, signals from other organs, such as the brain and body muscle, could accelerate intestinal immunosenescence during aging. However, given that studies on age-related changes in other organs have thus far lagged behind research into the programs underlying intestinal aging, solving this problem is currently difficult. Because aging is a stressful condition that causes rapid functional defects in several determinants of the instigation of ISC proliferation, accompanied by the emergence of inflammation or cancer-related phenotypes, it is also of interest to understand how the aging intestine signals to the peripheral organs, contributing to systemic immunosenescence. Several other questions remain for future studies. For example, much progress has been made in our understanding of the role of adult hemocytes in intestinal regeneration. Because most adult hemocytes reside near the middle midgut (Ayyaz et al., 2015), one of the most important questions in this field is whether hemocyte-expressed signals also play a multitude of physiological roles in the adult *Drosophila* “stomach.” Additionally, given that the *Drosophila* MTs possess specific receptors that recognize several hormones produced by the intestinal endocrine system (Johnson et al., 2005; Söderberg et al., 2011; Reiff et al., 2015), such as Pdf-R, TK-R, DH31-R, and DH44-R, a more thorough characterization of functional differences among these receptors will provide a necessary link to clarify how the MTs receive different signals from the intestine to regulate the basic biological processes of the renal system.

Finally, the similarities between mammals (such as humans) and *Drosophila* are not limited to their organic elements but are also evident in their interorgan communication signals (Droujinine and Perrimon, 2016). First, several organs in *Drosophila* have human equivalents, including the posterior midgut (human small intestine), fat body (human adipose tissue) and MTs (human kidney) (Droujinine and Perrimon, 2016). Second, it is remarkable that most of the signals identified to date in *Drosophila* organ-organ communication axes have counterparts that also regulate communication between organs in humans, including myoglianin (human growth differentiation factor (GDF)-11) and Upd2 (human leptin) (Droujinine and Perrimon, 2016). Furthermore, the lessons learned from *Drosophila* on interorgan communication have striking parallels with the situation in humans. For example, as discussed earlier, feeding causes Upd2 production in the fat body, stimulating the JAK-STAT signaling pathway in GABAergic neurons (Rajan and Perrimon, 2012). Similarly, nutrient-mediated adipokine leptin production in adipose tissue acts on a neuroendocrine organ (brain) via the leptin receptor in humans (Droujinine and Perrimon, 2016). However, one difference between the two species is that humans possess bones, blood vessels and adaptive immunity, which do not exist in *Drosophila* (Droujinine and Perrimon, 2016). In conclusion, research into organ-organ communication continues to be a rich and diverse field, and it is likely that data obtained from thorough studies investigating organ-to-organ communication in *Drosophila* will facilitate the discovery of similar mechanisms in humans.

## Author contributions

QL contributed to the writing of this review article. LJ approved the final version of the manuscript. QL and LJ are accountable for the entire contents.

## Funding

The project was supported by the grant from the National Natural Science Foundation of China (31270923) and the Fundamental Research Funds for the Central Universities (2572016EAJ4).

### Conflict of interest statement

The authors declare that the research was conducted in the absence of any commercial or financial relationships that could be construed as a potential conflict of interest.
